# Kounis Syndrome: An Entity One Should Not Forget

**DOI:** 10.7759/cureus.100128

**Published:** 2025-12-26

**Authors:** Joana Paulo, Daniela Marto, Filipa Figueiredo, Jéssica Oliveira, Mariana Costa

**Affiliations:** 1 Internal Medicine, Hospital Professor Doutor Fernando Fonseca, Lisbon, PRT; 2 Internal Medicine, Hospital Professor Doutor Fernando Fonseca, Amadora, PRT

**Keywords:** acute coronary syndrome, allergy and anaphylaxis, allergy prevention, drug-related side effects and adverse reactions, kounis syndrome (ks)

## Abstract

Kounis syndrome is an underreported diagnosis, even though it is a frequent pathology. It is an acute coronary syndrome that can present in the setting of an allergic or an anaphylactic reaction that could be triggered by the use of drugs. We present the case of a patient with a known medical history of drug allergies (but not of anaphylaxis), who presented to the emergency department with complaints of headache. After the administration of a painkiller, she developed hypotension, syncope, elevation of troponin, and transitory changes in the ST segment of the electrocardiogram.

## Introduction

Kounis syndrome is an acute coronary syndrome that happens in the setting of allergic or anaphylactic reactions and can be classified into three types: vasospastic angina (type 1), myocardial infarction (type 2), and stent thrombosis (type 3) [1,2]. This clinical entity goes frequently undiagnosed, namely in emergency care setting, especially due to the various ways it can present.

The pathogenesis of this condition is related to the role of the inflammatory mediators released by the mast cells in the cardiac vasculature, which can result in coronary vasoconstriction [1].

This condition can be triggered by many factors, namely by drugs, which is very frequent in healthcare settings. The most commonly associated drugs are the non-steroidal anti-inflammatory drugs and analgesics, but also antibiotics, proton pump inhibitors, and intravenous contrast. Other triggers have been reported, such as animal bites, food, and even cardiac stents [1,3]. The clinical presentation is varied, and it can present as shock, cardiac arrest, or, at early stages, just as headache, nausea, malaise, or vomiting. Extreme sweating, cold extremities, hypotension, and tachycardia are the most common signs. Even though it is an allergic reaction, it does not always present with skin changes, and so, even in the absence of this sign, the diagnosis should not be discarded. The initial approach consists of administering intravenous adrenaline and antihistamines. However, one should not forget that the use of adrenaline may aggravate myocardial ischemia [1,4].

There are no generally accepted guidelines for the treatment of this condition, and the number of reported cases is still low. The approach is, therefore, guided based on clinical case reports.

## Case presentation

This case is about a 75-year-old woman with a known medical history of hypertension, high cholesterol, and known allergies to paracetamol, tramadol, and etodolac, having previously had rash and nausea upon the use of these drugs.

**Figure 1:**
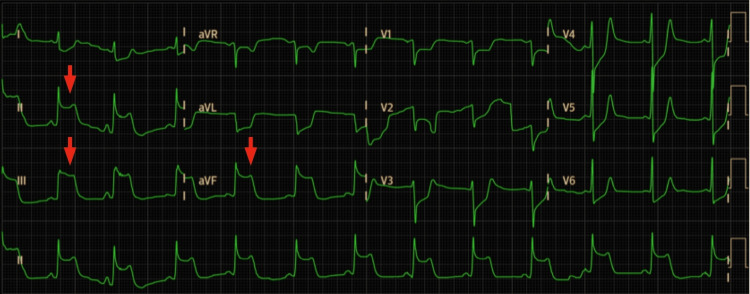
First ECG The red arrows show the ST-segment elevation in leads II, III, and aVF. ECG, electrocardiogram.

The patient came to the emergency department complaining of a severe headache and dizziness lasting for two days. She was initially treated with intravenous metamizole with no immediate reaction. About 45 minutes after, she had a syncope with spontaneous recovery but showed sustained hypotension. The electrocardiogram (ECG) showed ST segment elevation on the inferior leads (Figure 1), and the blood work collected at that time showed elevated troponin T of 84,30 ng/L (reference value <55 ng/L) (Table 1). 

**Table 1:** Troponin levels at the time of symptoms onset

Parameter	Reference value	Patient results
Troponin	<55 ng/L	84,30 ng/L

**Figure 2:**
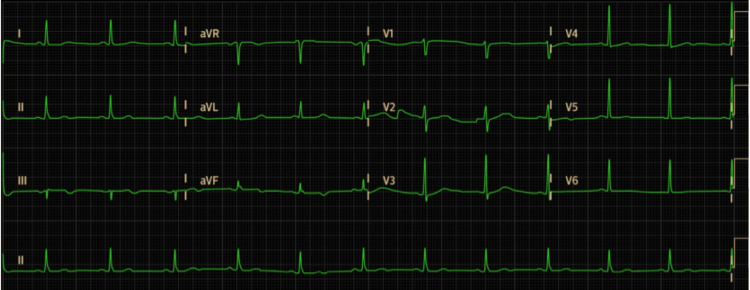
Second ECG ECG after resolution of symptoms shows normalization. ECG, electrocardiogram.

Assuming myocardial infarction as the diagnosis, the patient underwent coronary angiography, which did not show a thrombus or obstructive disease in need of reperfusion or intervention. After this procedure, the patient developed a skin rash. It was at this moment that Kounis syndrome as a diagnosis was considered. The patient was then treated with intravenous corticosteroids and antihistamines, with rapid improvement of the symptoms. Adrenaline was not used as, at that time, the patient did not present symptoms consistent with anaphylactic shock and due to the risk of myocardial ischemia. The ECG was repeated a few hours later, showing none of the previous changes (Figure 2). Troponin levels also showed a consistent decrease.

For the rest of the hospital stay, the patient had no other periods of hypotension, nausea, headache, chest pain, or sweating. Upon discharge, she was fully asymptomatic. She was referred to an outpatient consultation by allergology and internal medicine. The patient was instructed to avoid taking metamizole, tramadol, paracetamol, and etodolac and always mention this episode when receiving any kind of treatment.

## Discussion

This case highlights an infrequent diagnosis, not because of its prevalence being low, but because it goes frequently undiagnosed and reported. The presenting symptoms, with headache and nausea being non-specific, made the initial evaluation more challenging. However, allergic reactions may in fact manifest in early stages, either prodromic symptoms such as vasovagal, skin changes, dyspnea, abdominal pain, nausea, or headache [2].

Because the patient was female, older, and with a known medical history of high cholesterol, the hypothesis of myocardial infarction with atypical presentation was a possibility, particularly in the setting of the later symptoms with syncope and sustained hypotension. 

It will remain undetermined if the presenting complaints could already be attributed to an allergic reaction, later exacerbated by the use of intravenous contrast, or if the drug was the culprit alone. Moreover, the use of contrast for the angiography may have had a determinant role in the exacerbation of the symptoms [1].

Due to the rapid improvement of the initial ECG findings, the consistent decrease in troponin levels, the improvement of the symptoms, and the result of the coronary angiography, one can conclude that this was a case of vasospastic (type 1) Kounis syndrome [1,3]. Because this condition was not initially considered, tryptase levels were not measured. Serum tryptase is a biomarker for the evaluation and diagnosis of mast cell disorders, and so it would have been useful in confirming the diagnosis.

## Conclusions

Kounis syndrome remains underdiagnosed and poses a challenge, especially in an emergency care setting where rapid decisions must be made. The presented case demonstrates that unspecific symptoms followed by hemodynamic and electrocardiographic changes suggestive of acute coronary syndrome may be a hypersensitivity reaction mediated by mast cells, in this case, probably triggered or exacerbated by the use of pharmacological agents.

It also demonstrates the need for cautious use of drugs and the importance of keeping adequate vigilance of the patients while medication is being administered, especially concerning patients with previously known drug allergies.
